# Double Cortex Syndrome: An Unusual Cause of Seizures

**DOI:** 10.7759/cureus.63507

**Published:** 2024-06-30

**Authors:** Soumia Nedday, Yahya Naji, Mariam Assardoun, Sara Laadami, Nawal Adali

**Affiliations:** 1 Department of Neurology, Agadir University Hospital, Agadir, MAR; 2 Department of Neurology, Neurosciences Innovation Cognition Ethique (NICE) Research Team, Rein Endocrinologie Gastroentérologie Neurosciences Ethique (REGNE) Research Laboratory, Faculty of Medicine and Pharmacy, Ibn Zohr University, Agadir, MAR; 3 Department of Neurology, Agadir Medical School, Agadir, MAR

**Keywords:** antiseizure medication, double cortex, epilepsy, heterotopia, seizure

## Abstract

Gray matter heterotopia (GMH) is caused by abnormal neuronal migration during brain development. Subcortical band heterotopia (SBH), or double cortex, is a rare variant of GMH that mainly affects female patients with epilepsy (PWE) with different degrees of mental retardation. We present the case of a 25-year-old woman who was admitted to the neurology department of our tertiary hospital with generalized tonic-clonic seizures. Her mother had a normal antenatal period and a history of labor. There was a history of immediate crying and normal appearance, pulse, grimace, activity, and respiration (APGAR) scores. She had delayed milestones, which affected various categories of child development. Physical examination revealed a global developmental delay. Laboratory values, including complete blood count, serum calcium, and arterial blood gas tests, were all within normal limits. An EEG showed significant abnormalities suggestive of epilepsy. An MRI of the brain showed a continuous band of gray matter located deep and parallel to the cortex in both cerebral hemispheres, suggesting double cortex syndrome (DCS).

## Introduction

Malformation of cortical development encompasses a diverse range of abnormal cortical formations with various morphological abnormalities, genetic causes, and clinical presentations. The advent of brain MRI has greatly contributed to the precise determination of specific morphologies of cortical malformations [1]. Gray matter heterotopia (GMH) is a set of brain malformations characterized by the localization of neurons at abnormal positions. These disorders can present as clusters of neurons along the walls of the lateral ventricle, known as "subependymal heterotopia," nodules formed in the white matter, termed "focal subcortical heterotopia," or as bands of subcortical heterotopic neurons forming between the lateral ventricle and cerebral cortex, referred to as "subcortical band heterotopia (SBH) or double cortex syndrome (DCS)" [2,3].

SBH is a rare disorder of cortical migration, with sex-linked inheritance (90% of cases are females), and commonly occurs because of mutations in the doublecortin (DCX) gene, located on the long arm of chromosome X [4]. It presents with developmental delay and seizures, which may be refractory [4]. Symptom severity is related to the thickness of the heterotopic band and the associated cortical abnormalities [5]. MRI detects neuronal migration abnormalities and helps neurologists with medical care [5]. We report a case of SBH in a young woman who presented with developmental delay and refractory seizures, along with a review of the literature.

## Case presentation

A 25-year-old woman with early infantile developmental and epileptic encephalopathies was admitted to the neurology department. Her birth was at term, with a normal delivery. She had delayed milestone acquisition, affecting language, social interaction, cognition, and intellectual skills, resulting in severe intellectual disability. She was not independent and always depended on her mother. Since her first three months of life, she had multiple seizure types, including generalized tonic-clonic, atonic, and focal seizures with visual hallucinations at a frequency of three times a week. She was taking 500 mg of lamotrigine and 1,500 mg of sodium valproate daily without improvement.

**Figure 1:**
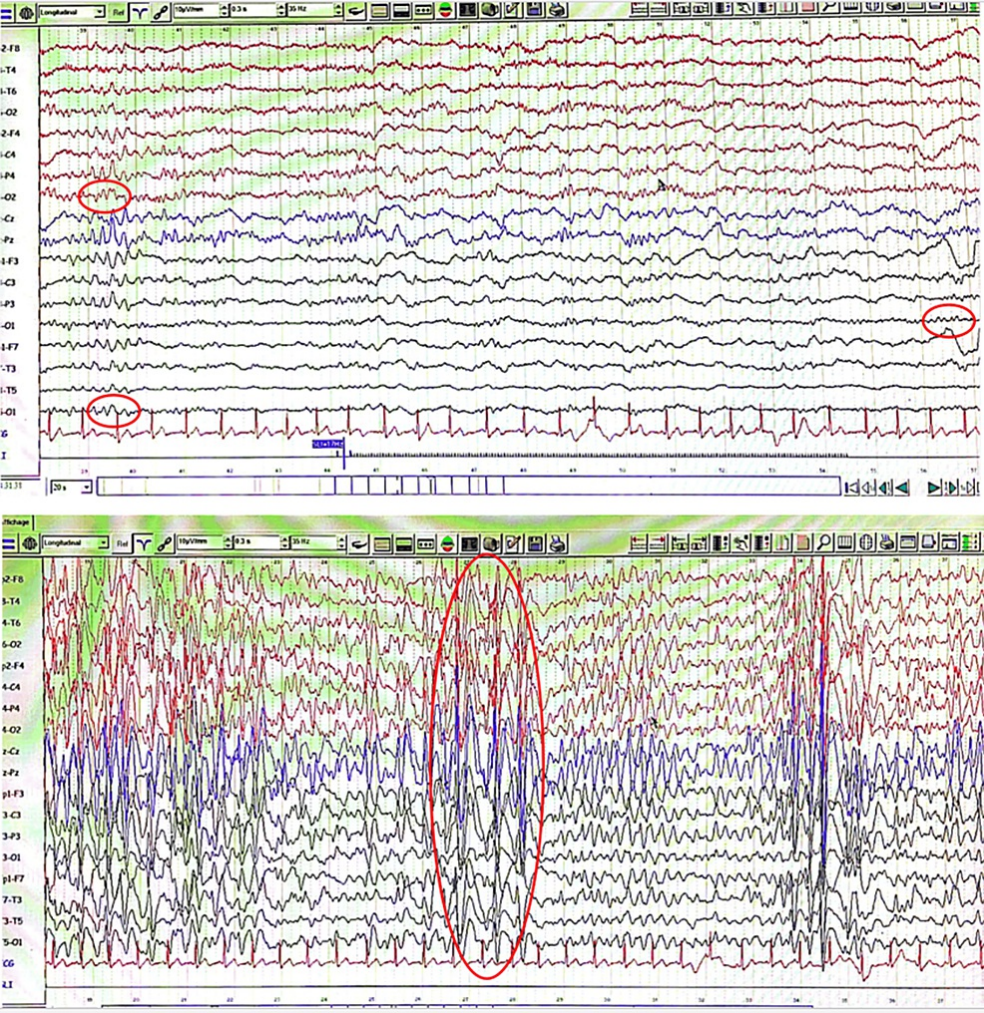
An EEG in the bipolar montage showing diffuse slowing with a posterior-dominant theta rhythm (5-7 Hz) on eye closure (circled), along with generalized spikes and slow waves (circled).

A physical examination revealed a conscious patient with normal tone, muscle bulk, strength in her upper and lower extremities, and exaggerated deep tendon reflexes in the four limbs. She showed profound cognitive impairment with a reduced mini-mental state examination (MMSE) score of 9/23 (she was illiterate). An ophthalmological examination revealed no abnormalities, including normal visual acuity. The anterior segment and fundus examination results were normal. Laboratory test results, including complete blood count and serum calcium level, were normal. Her EEG revealed diffuse slowing with a posterior-dominant theta rhythm along with generalized spikes and slow waves (Figure 1).

**Figure 2:**
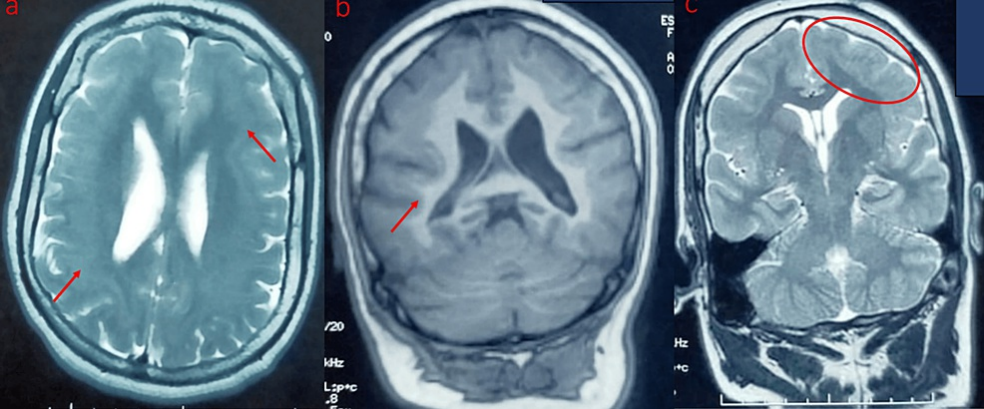
MRI brain axial T2 (a), coronal T1 (b), and coronal T2 (c) sequences showing characteristic subcortical heterotopic bands of gray matter (straight red arrows) with location and signal intensity parallel to the pachygyric overlying cortex (red circle).

A brain MRI revealed broad, symmetrical bilateral bands in the white matter, parallel to the cerebral cortex, and of similar signal intensity to that of the gray matter, with predominantly posterior pachygyria (Figure 2). A laboratory evaluation of complete blood cell count, biochemical tests (blood urea nitrogen, creatinine, sodium, potassium, phosphocalcic, and glucose), and thyroid tests were normal. For seizure management, levetiracetam was initiated at a dose of 1,000 mg/day, along with lamotrigine (500 mg/day) and clobazam (20 mg/day). Three months later, the clinical outcome showed a decrease in seizure frequency from three times per week to twice per month.

## Discussion

SBH is a rare type of cortical malformation characterized by bands of gray matter found bilaterally in the subcortical white matter [5]. However, the exact prevalence remains uncertain [6]. The etiopathogenesis of this malformation remains unclear. SBH can be sporadic or familial, with transmission linked to the X chromosome [7]. Considered a mild form of lissencephaly, this disorder is primarily observed in women and typically results in varying levels of intellectual disability. Almost all of the affected develop epilepsy [8]. The onset of seizures can occur at any age, mainly in the first decade, but they can sometimes be delayed until the second or third decade of life [9]. Seizure types include mixed partial, tonic-clonic, atypical absence, or drop attacks [10]. Our patient presented with generalized tonic-clonic seizures with a temporal focal onset and atonic seizures at a fairly early life stage.

There is no specificity for EEG results described in the literature. Some authors have found focal or multifocal spikes and waves in centrotemporal regions. Others describe diffuse slow waves, generated polyspikes and waves, or diffuse spikes [6]. The EEG of our patient revealed generalized and slow-wave spikes. An MRI allows the visualization of subcortical bands of gray matter, which appear as a layer of gray matter arranged symmetrically and circumferentially with smooth margins under the cortex. It is separated from the overlying cortex and underlying ventricle by white matter [11,12]. The risk of severe disability and developmental delay increases as the band thickness of heterotopic neurons increases [13]. The association of SBH with lissencephaly, pachygyria, or agyria has been reported in the literature [14]. In our case, an MRI showed an SBH associated with predominantly posterior pachygyria.

Most people with SBH have mutations in the DCX or lissencephaly-1 (LIS1) gene. The latter is located at 17p13.3, whereas DCX is located at Xq24 [15,16]. Since DCX is carried on the X chromosome of males, DCX mutations generally cause classic lissencephaly in males, whereas females have SBH [17]. Unfortunately, genetic testing of our patient could not be performed because of a lack of resources.

Epilepsy in patients with SBH may be refractory to pharmacological treatments. However, a similar case report described a good response to polytherapy, including sodium valproate, levetiracetam, and clobazam [9]. Other authors used lamotrigine, sodium valproate, and levetiracetam, with good control of epilepsy in the following six months [18]. Our patient was treated with lamotrigine, levetiracetam, and clobazam with good stabilization. Non-pharmacological treatment was based mainly on corpus callosotomy and temporal lobectomy, with a few published cases. However, we cannot conclude with certainty the best surgical option to treat refractory epilepsy in patients with SBH, given the limited number of cases reported in the literature [19].

## Conclusions

SBH and DCS are rare variants of GMH. This typically causes varying degrees of psychomotor retardation and epilepsy. In our case, we highlight the importance of brain MRI in the face of any epileptic encephalopathy syndrome, which helps in making the diagnosis and searching for associated malformations to establish adequate treatment and allow the patient to achieve good social integration. Moreover, we emphasize the importance of genetic analysis and genetic counseling given the familial nature of this syndrome. Accessibility of genetic testing remains a major issue in many parts of the world and precludes optimal personalized approaches for DCS and related disorders.
